# Prediction of Submucosal Invasion for Gastric Neoplasms in Endoscopic Images Using Deep-Learning

**DOI:** 10.3390/jcm9061858

**Published:** 2020-06-15

**Authors:** Bum-Joo Cho, Chang Seok Bang, Jae Jun Lee, Chang Won Seo, Ju Han Kim

**Affiliations:** 1 Medical Artificial Intelligence Center, Hallym University Medical Center, Anyang 14068, Korea; donaldaq@gmail.com; 2 Department of Ophthalmology, Hallym University Sacred Heart Hospital, Anyang 14068, Korea; 3 Division of Biomedical Informatics, Seoul National University Biomedical Informatics (SNUBI), Seoul National University College of Medicine, Seoul 03080, Korea; juhan@snu.ac.kr; 4 Institute of New Frontier Research, Hallym University College of Medicine, Chuncheon 24253, Korea; iloveu59@hallym.or.kr; 5 Department of Internal Medicine, Hallym University College of Medicine, Chuncheon 24253, Korea; 6 Institute for Liver and Digestive Diseases, Hallym University, Chuncheon 24253, Korea; 7 Division of Big Data and Artificial Intelligence, Chuncheon Sacred Heart Hospital, Chuncheon 24253, Korea; 8 Department of Anesthesiology and Pain Medicine, Hallym University College of Medicine, Chuncheon 24253, Korea

**Keywords:** artificial intelligence, convolutional neural networks, endoscopy, gastric neoplasms

## Abstract

Endoscopic resection is recommended for gastric neoplasms confined to mucosa or superficial submucosa. The determination of invasion depth is based on gross morphology assessed in endoscopic images, or on endoscopic ultrasound. These methods have limited accuracy and pose an inter-observer variability. Several studies developed deep-learning (DL) algorithms classifying invasion depth of gastric cancers. Nevertheless, these algorithms are intended to be used after definite diagnosis of gastric cancers, which is not always feasible in various gastric neoplasms. This study aimed to establish a DL algorithm for accurately predicting submucosal invasion in endoscopic images of gastric neoplasms. Pre-trained convolutional neural network models were fine-tuned with 2899 white-light endoscopic images. The prediction models were subsequently validated with an external dataset of 206 images. In the internal test, the mean area under the curve discriminating submucosal invasion was 0.887 (95% confidence interval: 0.849–0.924) by DenseNet−161 network. In the external test, the mean area under the curve reached 0.887 (0.863–0.910). Clinical simulation showed that 6.7% of patients who underwent gastrectomy in the external test were accurately qualified by the established algorithm for potential endoscopic resection, avoiding unnecessary operation. The established DL algorithm proves useful for the prediction of submucosal invasion in endoscopic images of gastric neoplasms.

## 1. Introduction

Surgical resection has been the standard treatment method for gastric neoplasms. However, the advancement of endoscopic techniques over recent years has led to a better understanding of various therapeutic indications and outcomes, allowing for the replacement of the classical surgery with endoscopic resection, in a subset of gastric cancer patients who meet certain specific criteria [1,2]. The commonly accepted consensus allows for endoscopic resection with curative intent, in cases of gastric neoplasms without lymph node metastasis (LNM). Therefore, the indication for endoscopic resection is based on a combination of factors associated with a low LNM rate, which are retrospectively assessed in surgically resected specimens [3,4]. These factors include the depth of invasion, its specific size, and morphological and histological lesion properties [5]. Of note, the endoscopic resection is a greatly preferred method, owing to its minimal invasiveness and quick patient recovery [2].

The large depth of invasion is one of the most important risk factors for LNM. Gastric neoplasms confined only to the mucosa or superficial submucosa (within 500 µm) are potential candidates for endoscopic resection. Therefore, precise assessment of the depth of invasion is essential for the determination of an optimal treatment strategy for gastric cancers. Determination of invasion depth is usually based on gross tumor morphology observed in endoscopic images or on endoscopic ultrasound data. However, these methods are not always precise and may result in inter-observer variability. Previous studies indicated that endoscopic ultrasound and gross morphology method are equally accurate in estimating the invasion depth of early gastric cancers (EGCs), and that expert endoscopists can correctly determine the invasion depth of approximately 70–80% tumors [6,7]. Since it is difficult to precisely predict the depth of invasion of lesions prior to their resection, patients need to be informed about the possibility of additional surgery or endoscopic resection, in case curative resection is not achieved [8].

Convolutional neural network (CNN) is a deep-learning (DL) algorithm widely used in the classification of medical images and has been increasingly adopted in clinical practice [9,10,11,12,13]. CNN is a type of specific artificial neural networks that consists of multiple convolutional and pooling layers and image filters (kernels), to automatically extract certain features from images enabling an overall classification [14,15]. CNN classifies images by performing a specialized type of linear operation with convolutional matrices, which are small grids of parameters, and by reducing dimensions through the max pooling process [14,15]. For example, it reduces the image dimensions from 4 × 4 pixels to 2 × 2, by using filters to select the largest value from convolutionally operated values, and by reducing the dimensions through max pooling. A certain feature that characterize the images extracted from the convolutional and pooling layers is applied to the input value of the final layer of the fully-connected neural network, and serves as the image classification through the activation functions such as softmax regression [15].

Several studies presented CNN-based DL algorithms, which classify the invasion depth of EGCs [16,17,18]. However, these algorithms are only used after the definite diagnosis of gastric cancers, which is limited in real clinical practice, owing to various types of gastric neoplasms. Optical diagnosis with visual inspection has a putative nature, and a definitive diagnosis only can be made after resection of the lesions via histologic examination. Therefore, the study should include all neoplastic lesions with potential for resectability, and this study aimed to establish a DL algorithm for the prediction of the depth of invasion in endoscopic images of gastric neoplasms.

## 2. Materials and Methods

### 2.1. Collection of Data

Consecutive patients who were found to have any type of gastric neoplasm during upper gastrointestinal endoscopy between 2010 and 2017 at Chuncheon Sacred Heart Hospital were enrolled. The aim of endoscopic examinations and detailed procedures are described in our previous report [19]. All the neoplasm-suspected lesions that were resected using either the endoscopic resection (endoscopic mucosal resection or endoscopic submucosal dissection technique (ESD)) or surgical resection were included. Pathological assessment of each lesion was carried out by two pathologists. Samples defined as tumors were cross-checked by yet another pathologist in the Chuncheon Sacred Heart hospital [19].

White-light imaging (WLI) endoscopy data from gastric lesions with pathological confirmation were retrieved from the database of Chuncheon Sacred Heart hospital in JPEG format, with a minimum resolution of 640 × 480 pixels. Images with poor quality or low resolution disabling their proper classification (defocusing, artifacts, shadowing, etc.) were excluded, as previously described [19]. Eventually, a total of 2899 images from 846 patients were included in the study. This study was approved by the Institutional Review Board of Chuncheon Sacred Heart Hospital (number: 2018-07-003), and was conducted in accordance with the Declaration of Helsinki. IRB approval number: 2018-07-003.

### 2.2. Construction of Dataset

All images were divided into two groups, namely the mucosa-confined group and the submucosal invasion group, based on pathologic records reviewed by an experienced endoscopist (C.S.B.) [19]. These were also sub-classified into four classes based the following clinical properties: low-grade dysplasia, high-grade dysplasia, EGC, and advanced gastric cancer (AGC). EGC was defined as an adenocarcinoma confined to the mucosa or submucosa, irrespective of LNM. AGC was defined as an adenocarcinoma infiltrating beyond the submucosal layer, irrespective of LNM. The whole dataset was divided into two datasets, namely the training and the internal test datasets, with a ratio of 9:1. The division was done by random sampling using patients’ IDs as sample descriptors. The same class images from a single patient were assigned together to either the training set or the test set. The training dataset was then divided into a proper training dataset and a validation dataset for hyper-parameter tuning, with a ratio of 8:1, with three different random seeds.

An external test dataset was constructed through the collection of images from consecutive patients who underwent upper gastrointestinal endoscopy at the Chuncheon Sacred Heart Hospital between 2019 and 2020. The collection period was exclusive from that of the internal test dataset or the training datasets.

### 2.3. Preprocessing of Datasets

After construction of the datasets, the submucosa-invaded group in the training dataset was augmented to overcome the data imbalance, as the mucosal group was approximately twice as big. Another copy of the submucosal invasion group images was created by rotating the original images by 90 degrees. After data augmentation, the number of submucosa-invaded images was doubled, and became similar to that of mucosa-confined images in the training dataset. Next, the whole training dataset was amplified four times by horizontal flipping and/or vertical flipping.

Following this, all images of the training dataset were resized into 480 × 480 pixels. Finally, the training images were normalized, using linear transformation, with the means and standard deviations known for the ImageNet datasets in terms of three RGB channels.

### 2.4. Training of CNN Models

Two CNN models, pre-trained with the ImageNet Large Scale Visual Recognition Challenge dataset were adopted, namely the Inception-ResNet-v2 and the DenseNet−161 models. The details of the models were described previously (https://arxiv.org/abs/1602.07261 and https://arxiv.org/abs/1608.06993). The Inception-ResNet-v2 was selected for its high performance, despite the huge number of weights, and the DenseNet−161 was selected for its good performance with a lower number of parameters. The backbone of both CNN models was frozen as a fixed feature extractor, and the final fully connected layers were fine-tuned using the training dataset.

Binary cross entropy was used as the loss function. The Adam optimizer was adopted, and the initial learning rate was 1 × 10^−4^. The learning rate was reduced as 1/10 at every 10 epochs, until it reached 1 × 10^−7^, and the learning rate was maintained on that level. After training of 50 epochs, early stopping was applied with the patience of 50 epochs. The batch size was 16, and no dropout was used. 

A class activation map (CAM) was implemented onto the CNN models, to show the attention map of the established algorithms. The final few layers of the CNN architectures were replaced by a global average pooling and a softmax layer. The training was performed using the PyTorch platform on the hardware system, with NVIDIA GeForce RTX 1080 Ti graphics processing units, dual Xeon central processing units, 128 GB RAM, and a water-cooling system.

### 2.5. Main Outcome Measurements and Statistics

The primary readout was the accuracy of the models in discriminating whether the lesion was mucosa-confined or submucosa-invaded in endoscopic images. The secondary outcome was the discrimination performance for the EGC subgroup. The model performances were tested three times upon random test sets changes. The main evaluation metric was the area under the curve (AUC) and the maximized diagnostic accuracy. Additionally, sensitivity, specificity, positive predictive value, and negative predictive value were evaluated at the optimal threshold points, maximizing Youden’s index. The categorical variables are expressed as percentages with 95% confidence intervals (CIs), and continuous variables are presented as mean ± standard deviation. A *p* value of < 0.05 was considered as statistically significant. All the analyses were performed using R software version 3.6.3 (The R Foundation for Statistical Computing, Vienna, Austria) and Python programming language (Python Software Foundation, version 3.7.5) with Scikit-learn package version 0.20.3.

## 3. Results

### 3.1. Composition of Datasets

A total of 2899 images from 846 patients were analyzed in this study. Among them, the images of submucosa-invaded lesions accounted for 34.5% (*n* = 999), and that of mucosa-confined lesions for 65.5% (*n* = 1900). In the internal test set, 309 images from 85 patients were included, among which 102 images from 27 patients were submucosa-invaded lesion images. The external test set comprised 206 images from 197 patients, and the submucosa-invaded lesion images accounted for 38.8% (*n* = 80). Table 1 describes the baseline composition of enrolled images in the training, internal testing, and external testing datasets.

### 3.2. Prediction of Submucosal Invasion in any Given Gastric Neoplastic Lesions

**Figure 1:**
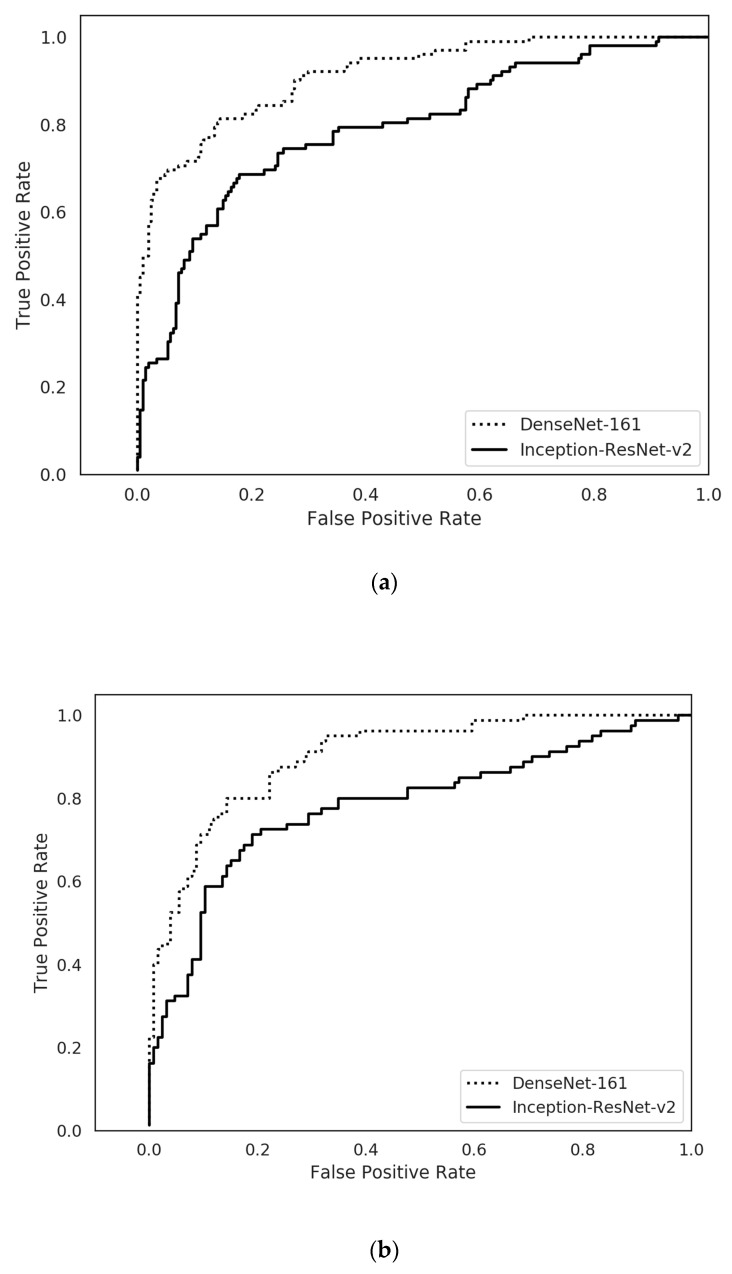
Receiver operating characteristic curve of the best performance model. (**a**): internal test, (**b**): external test.

Table 2 describes the performance of CNN models in the internal test dataset. The mean AUC and accuracy of the Inception-ResNet-v2 was 0.786 (95% CI, 0.779–0.793) and 77.4% (76.7–78.0%), respectively. The mean AUC and the accuracy of the DenseNet−161 was 0.887 (0.849–0.924) and 84.1% (81.6–86.7%), respectively. The receiver operating characteristic curve of the best performance model for internal test is presented in Figure 1a.

For the external test dataset, the mean AUC and accuracy of the Inception-ResNet-v2 were 0.769 (0.755–0.783) and 74.1% (71.0–77.2%), as presented in Table 3. The mean AUC and the accuracy of the DenseNet−161 were 0.887 (0.863–0.910) and 77.3% (75.4–79.3%), respectively. The receiver operating characteristic curves of the best performance model for external test is presented in Figure 1b.

**Figure 2:**
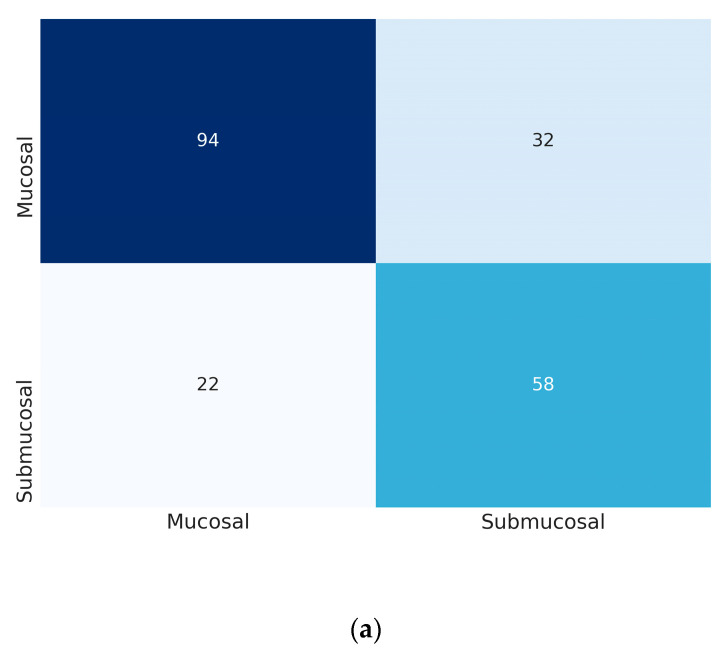

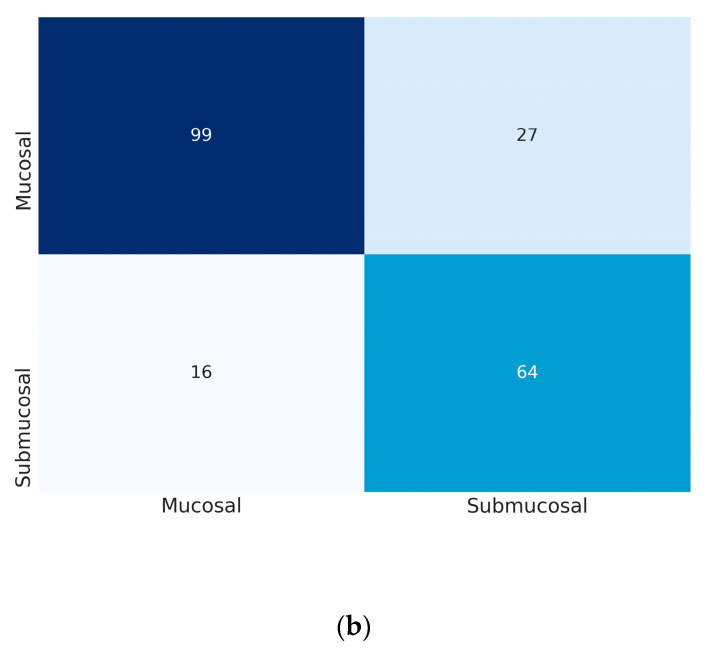
Confusion matrix of the best performance model in the external test. (**a**): the Inception-Resnet-v2, (**b**): the DenseNet−161. The vertical axis is true label and the horizontal axis is predicted label.

At the fitting point maximizing Youden index, the mean sensitivity and specificity of the model in the external test dataset were 72.5% (72.5–72.5%) and 74.3% (73.0–75.7%) by the Inception-Resnet-v2, and 80.4% (79.6–81.3%) and 80.7% (78.5–83.0%) by the DenseNet−161, respectively (Table 3). The positive predictive value and the negative predictive value of the DenseNet−161 were 72.6% (70.1–75.1%) and 86.6% (85.9–87.4%), respectively. The confusion matrices of the best performance model are presented in Figure 2.

### 3.3. Prediction of Submucosal Invasion in Subgroup of EGCs

Only a subgroup of EGCs was distributed in both the mucosa-confined and the submucosal invasion group as presented in Table 1, so the subgroup analysis of the performance was carried out. In the internal test dataset, the AUC and accuracy of the models in the prediction of submucosal invasion of EGC were 0.694 (0.607–0.781) and 71.4% (67.1–75.8%) by the DenseNet−161, respectively (Table 2).

In the external test dataset, which comprised 60 images, the mean AUC and accuracy of the DenseNet−161 were 0.747 (0.712–0.782) and 67.2% (64.4–70.1%), respectively (Table 3).

### 3.4. Clinical Simulation in the Application of DL Algorithm for the Determination of Therapeutic Strategy

**Figure 3:**
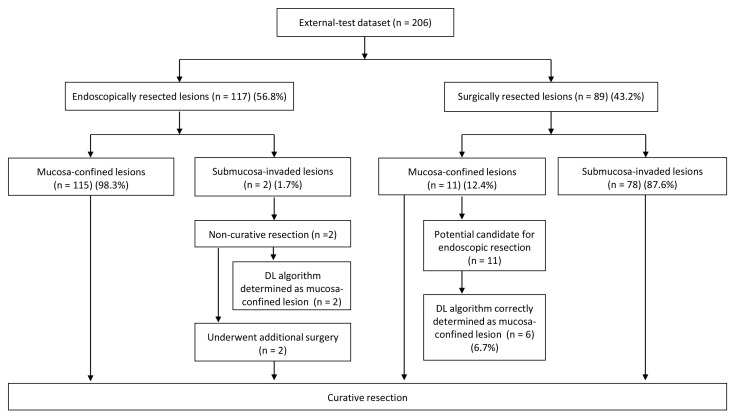
Clinical simulation in the application of the deep-learning algorithm for the determination of a therapeutic strategy of external test dataset; DL, deep-learning.

**Figure 4:**
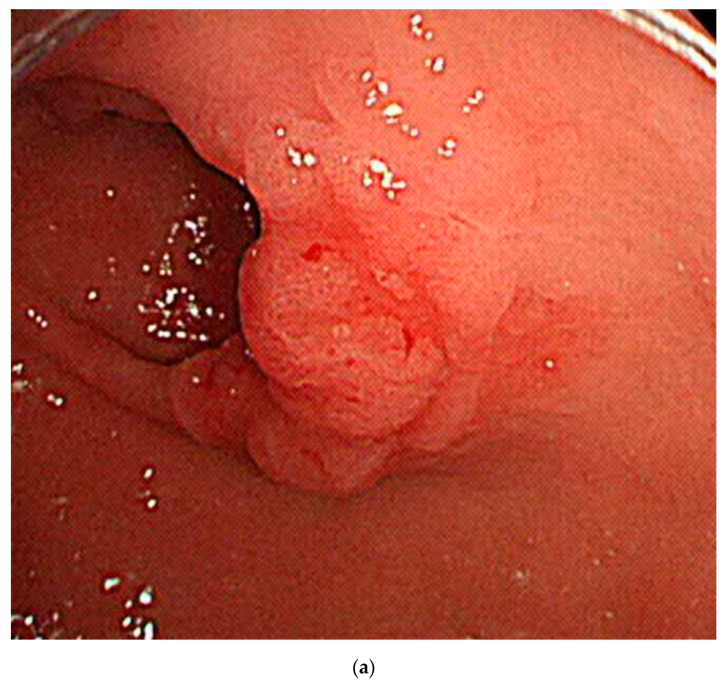

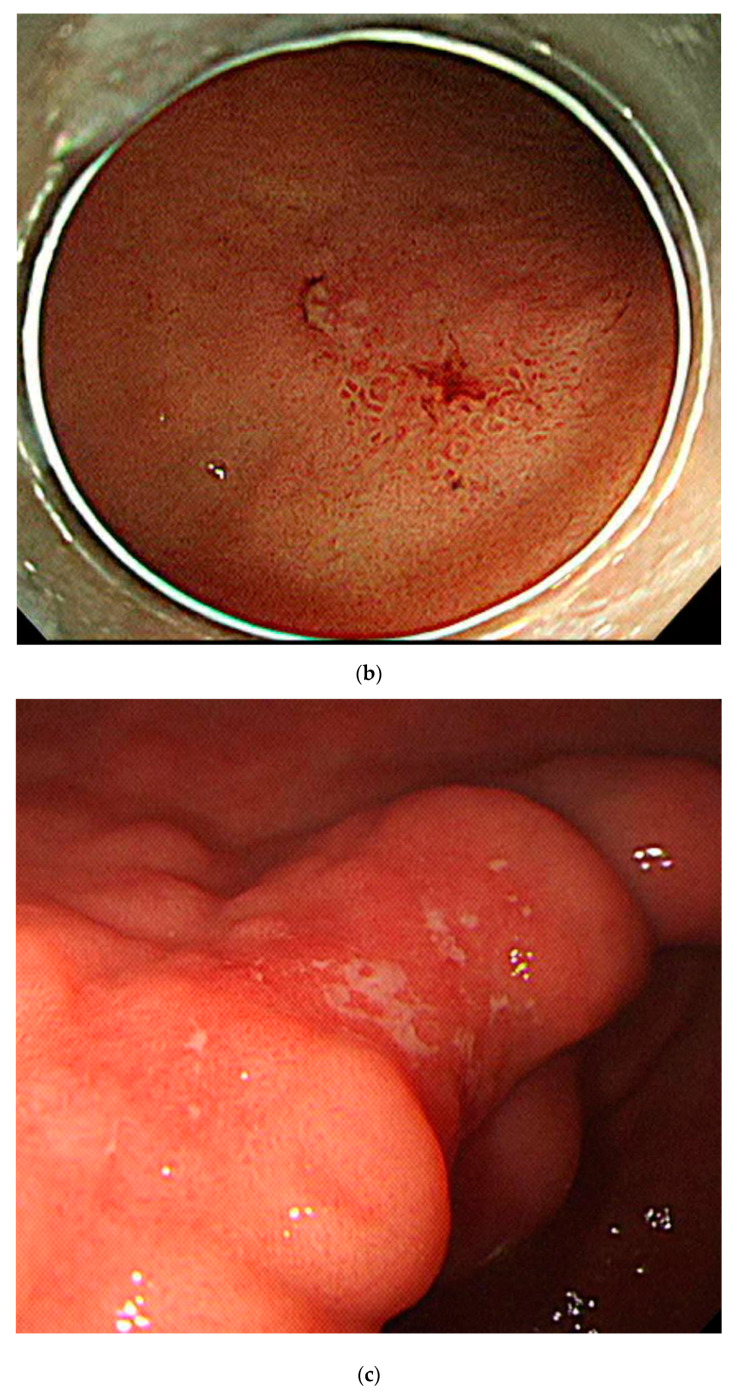

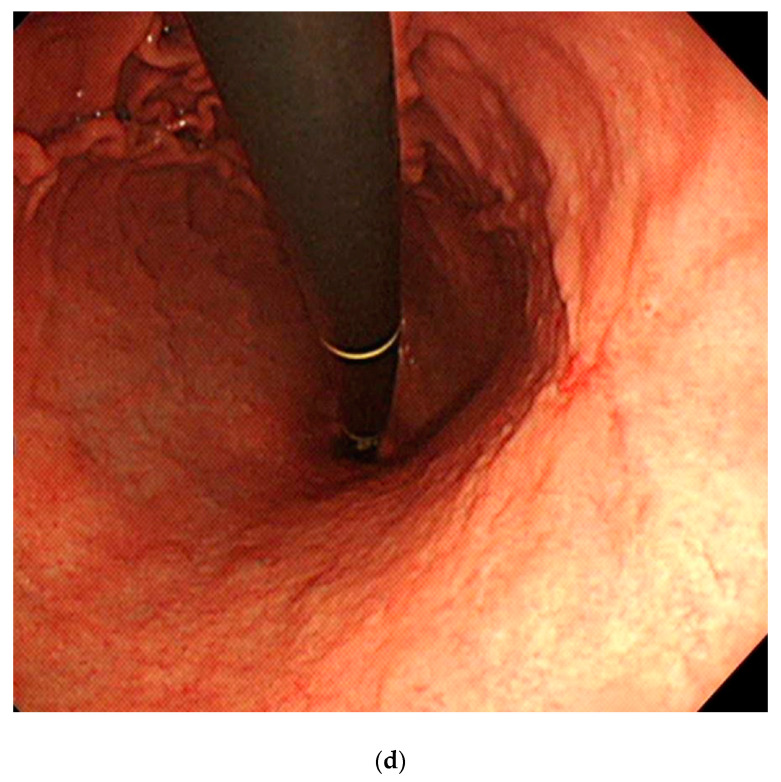
Representative images of correctly or incorrectly determined lesions by the deep-learning (DL) algorithm. (**a**): Wrong answer case by the DL algorithm in endoscopically resected lesions (moderately differentiated adenocarcinoma with SM2 invasion); (**b**): wrong answer case by the DL algorithm in endoscopically resected lesions (well differentiated adenocarcinoma with SM3 invasion); (**c**): correct answer case by the DL algorithm for endoscopic submucosal dissection (ESD) candidate in surgically resected lesions (mucosa-confined signet ring cell carcinoma with 1 cm diameter); (**d**): wrong answer case by the DL algorithm in surgically resected lesions (mucosa-confined poorly differentiated adenocarcinoma within expanded indication); DL, deep-learning; ESD, endoscopic submucosal dissection.

Clinical simulation was done for the lesions in the external test, assuming that the established algorithm was used to determine the therapeutic strategy choice: either the endoscopic resection or classical surgery. The choice was determined based on the depth of invasion in gastric neoplasms (Figure 3). Among 206 lesions, 117 lesions (56.8%) were endoscopically resected and 89 lesions (43.2%) were surgically resected in the external test dataset. For the endoscopically resected lesions, 115 lesions (98.3%) were confined to mucosa and two lesions (1.7%) invaded submucosa. All the mucosa-confined lesions achieved curative resection through endoscopic resection. However, two submucosa-invaded lesions did not achieve curative resection through ESD, and were subject to additional surgery for the definitive treatment. Like endoscopists who underestimated the invasion depth of these two lesions during their real clinical practice, the DL algorithm also diagnosed them as mucosa-confined lesions (wrong answer cases by DL algorithm in endoscopically resected lesions) (Figure 4a,b). For the surgically resected lesions, 78 lesions (87.6%) were submucosa-invaded and 11 lesions (12.4%) were mucosa-confined. Among the 11 mucosa-confined lesions that were potential candidates for endoscopic resection, six lesions (6.7%) were correctly classified as mucosa-confined lesions by the DL algorithm (correct answer case by DL algorithm for ESD candidate in surgically resected lesions) (Figure 4c). However, endoscopists determined these mucosa-confined lesions as submucosa-invaded lesions in their real practice, thus imposing surgical treatment as the therapeutic strategy. Figure 4d shows a representative case of surgically resected mucosa-confined lesion, which was also determined as submucosa-invaded lesion by the DL algorithm (wrong answer case by DL algorithm in surgically resected lesions).

### 3.5. Attention Maps

**Figure 5:**
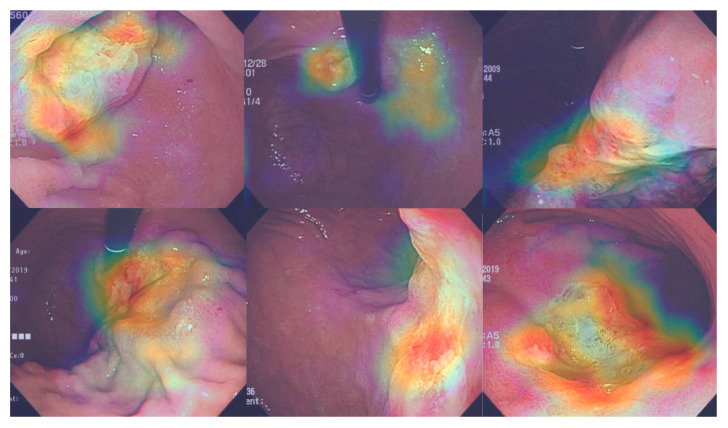
Representative images of the attention map.

Representative images of CAM for classifying submucosal invasion in endoscopic images are presented in Figure 5. The CAM images correctly demonstrated the submucosal neoplastic area, which is characterized by irregular/nodular surface, deep ulceration, fusion/clubbing fold, or irregular protrusion.

## 4. Discussion

In this study, we established a DL algorithm with a substantial diagnostic performance, in terms of discriminating the depth of invasion of gastric neoplasms (based on endoscopic white-light images) into a binary class (mucosa-confined vs. submucosa-invaded). The highest AUC value in the internal and external tests commonly reached 0.887. As the utilized learning model is data-driven and ‘black-box’-type in its nature, the only way to verify the real diagnostic performance of the DL algorithm was validation with an external test dataset [20]. Hence, we utilized an external test diagnostic verification method and observed the same algorithm performance value (AUC of 0.887), as during the internal testing.

Medical datasets tend to be heterogenous and imbalanced; hence, they are difficult to study in an artificial intelligence setting. In line with this, they are problematic when it comes to achieving optimized performance and reproducing the training performance of a DL algorithm in a real (clinical) setting. For instance, the fraction of ‘abnormal’ gastric lesions is normally much lower than that of ‘normal’ gastric lesions, making real-life lesion datasets significantly different from experimental datasets. We utilized consecutive samples derived from ‘real-life’ patients, so the fraction of mucosa-confined lesions was three times higher than that of submucosa-invaded lesions in these datasets (Table 1). Therefore, we had to adopt a data augmentation method in the submucosa-invaded lesion group, to minimize the imbalance in the training process. The learning rate scheduling and early stopping were also applied, to prevent overfitting (when the learning process tailors itself too much on the training data, and its performance cannot be reproduced on any new data). However, it is difficult to maintain the internal testing performance in the external testing dataset, because there is no optimal way to prevent the overfitting of a DL algorithm. In our study, the main reason for a comparable diagnostic performance in external testing was the improvement of the data imbalance. The proportion of submucosal invasion group was increased in the external testing, compared to that in the internal testing. The external testing in our study was not performed in a multicenter setting with large datasets, so the real value of the established algorithm could be verified in the future with clinical application studies.

Previous studies have established DL algorithms for the discrimination of the invasion depth of gastric cancers [16,17,18]. Kubota et al. established a deep neural network-based algorithm classifying the depth of gastric cancer invasion, irrespective of EGC or AGC. The discriminating accuracy was 77.2%, 49.1%, 51%, and 55.3% for stages T1–T4, respectively (the accuracy discriminating between T1a (mucosa-confined) and T1b (submucosa-invaded) was 68.9%) [16]. Zhu et al. also built a CNN-based algorithm for the discrimination of invasion depth in EGC or AGC, and reported a diagnostic accuracy of up to 89.16% [17]. Yoon et al. enrolled only EGC cases, and also constructed a CNN-based algorithm. In their study, the AUC for discriminating the depth of invasion was 0.851 [18].

Importantly, all these previous studies report internal testing accuracy or AUC, with no external testing verifications. Therefore, their reported diagnostic performances might be exaggerated, as external testing should always be performed when determining a learning model’s accuracy. Moreover, all previous studies assumed that a definitive diagnosis of gastric cancers was made prior to the application of their DL algorithms. However, clinical studies of gastric neoplasms reveal that the final pathology status is upgraded in 15.9% of cases, and downgraded in 6.9% of cases after ESD or surgery, as compared to the status defined upon an initial endoscopic biopsy (i.e., initial diagnosis of low-grade dysplasia on the endoscopic biopsy can be changed to the final diagnosis of EGC after ESD, thus upgrading the pathological status) [21]. Conclusively, such algorithms cannot be utilized in clinical practice (as not all lesions found during endoscopy are gastric cancers). The diagnostic performance of the established model in our study seems to be lower than that of the previous studies, which stems from discrepancies in the inclusion criteria.

Previous studies focused only on gastric cancers, which is not a realistic approach in clinical settings. Therefore, the per-class performance of EGC in our study is also lower than that of the other studies. Notably, as we only performed external testing of performance verification, a comparison of the diagnostic performance with other studies is not possible. Further training with the accumulation of EGC cases would definitely enhance the per-class performance observed in our study.

Although we did not test this established algorithm in a real clinical setting, we conducted a retrospective clinical simulation on an external testing dataset, categorized by a therapeutic strategy employed. Interestingly, our algorithm underestimated the invasion depth of two lesions (shallow erosion was noted in both cases), which had also been a mistake made by endoscopists prior to our study (Figure 4a,b). However, among the 11 mucosa-confined and surgically resected lesions, six lesions (6.7%) were correctly classified as mucosa-confined lesions by our DL algorithm, thus potentially avoiding unnecessary operation in 6.7% of patients in the clinical simulation. This indicates a clinical utility of our algorithm in the therapeutical decision-making process. This clinical simulation was not performed in a randomized and controlled manner, so more evidence through a randomized-controlled trial would elucidate the real value of this algorithm [22].

Despite its potential prognostic applicability, our study has several limitations. First, the utilized images were retrieved from a single medical center and their number was rather small. Second, the external testing dataset was also retrieved from a single medical center, and no multicenter validation was conducted. These two limitations are expected to be resolved by a prospective multicenter validation of the established algorithm. Third, the clinical utility of the algorithm was only confirmed in a retrospective clinical simulation. Therefore, a randomized study in a clinical setting would shed light on the real utility of this model.

In conclusion, the algorithm established in this study stems as a useful tool for the prediction of submucosal invasion events of gastric neoplasms, based on their endoscopic images. Thus, it has a potential clinical relevance during the choice of surgical strategy in gastric tumor patients.

## Figures and Tables

**Table 1:** Composition of datasets used in the development and testing of the deep-learning algorithm.

	Whole Dataset		Training Set		Internal Test Set		External Test Set	
	Number of Images	Number of Patients	Number of Images	Number of Patients	Number of Images	Number of Patients	Number of Images	Number of Patients
Overall	2899	846	2590	762	309	85	206	197
Mucosa-confined lesions	1900	580	1693	522	207	58	126	119
Low-grade dysplasia	727	233	630	205	97	28	68	66
High-grade dysplasia	421	131	390	123	31	8	21	21
EGC	752	230	673	205	79	25	37	37
Submucosa-invaded lesion	999	270	897	243	102	27	80	78
EGC	282	81	242	71	40	10	23	23
AGC	717	189	655	172	62	17	57	55

EGC, early gastric cancer; AGC, advanced gastric cancer.

**Table 2:** Diagnostic performance of the established algorithm classifying submucosal invasion in the internal test dataset.

Model	AUC	Accuracy (%)	Sensitivity (%)	Specificity (%)	Positive Predictive Value (%)	Negative Predictive Value (%)
Whole dataset						
Inception-Resnet-v2	0.786 (0.779–0.793)	77.4 (76.7–78.0)	72.5 (71.5–73.6)	72.9 (71.3–74.6)	56.9 (55.2–58.7)	84.4 (83.6–85.1)
DenseNet−161	0.887 (0.849–0.924)	84.1 (81.6–86.7)	78.8 (75.4–82.2)	80.0 (76.8–83.2)	66.1 (61.6–70.6)	88.4 (86.4–90.4)
EGC (*n* = 119)						
Inception-Resnet-v2	0.612 (0.599–0.626)	66.1 (65.0–67.2)	56.7 (55.0–58.3)	57.0 (54.1–59.9)	40.0 (38.3–41.8)	72.2 (70.9–73.4)
DenseNet−161	0.694 (0.607–0.781)	71.4 (67.1–75.8)	60.8 (54.9–66.7)	61.6 (54.2–69.0)	44.7 (37.7–51.7)	75.5 (70.4–80.6)

AUC, area under the curve; EGC, early gastric cancer.

**Table 3:** Diagnostic performance of the established algorithm for the prediction of submucosal invasion in the external test dataset.

Model	AUC	Accuracy (%)	Sensitivity (%)	Specificity (%)	Positive Predictive Value (%)	Negative Predictive Value (%)
Whole dataset						
Inception-Resnet-v2	0.769 (0.755–0.783)	74.1 (71.0–77.2)	72.5 (72.5–72.5)	74.3 (73.0–75.7)	64.2 (62.9–65.5)	81.0 (80.7–81.3)
DenseNet−161	0.887 (0.863–0.910)	77.3 (75.4–79.3)	80.4 (79.6–81.3)	80.7 (78.5–83.0)	72.6 (70.1–75.1)	86.6 (85.9–87.4)
EGC (*n* = 60)						
Inception-Resnet-v2	0.609 (0.572–0.647)	65.0 (61.7–68.3)	58.0 (55.1–60.8)	62.2 (56.9–67.5)	52.2 (40.8–63.6)	70.4 (67.3–73.4)
DenseNet−161	0.747 (0.712–0.782)	67.2 (64.4–70.1)	65.2 (65.2–65.2)	70.3 (67.2–73.4)	57.8 (55.3–60.3)	76.5 (75.7–77.2)

AUC, area under the curve; EGC, early gastric cancer.

## Abbreviations

DL	deep-learning
LNM	lymph node metastasis
EGC	early gastric cancer
CNN	Convolutional Neural Network
ESD	endoscopic submucosal dissection
WLI	white-light imaging
AGC	advanced gastric cancer
CAM	class activation map
AUC	area under the curve
CI	confidence interval
